# Autologous platelet-rich gel for refractory plantar ulcer following ethanol sclerotherapy of peripheral venous malformation: a Case Report

**DOI:** 10.3389/fbioe.2025.1700377

**Published:** 2025-12-18

**Authors:** Zhenyu Zhao, Shaohui Wu, Xiaoyan He, Yan Zhang, Xuemei Chen, Jie Tang

**Affiliations:** 1 Transfusion Department, Mianyang Central Hospital, School of Medicine, University of Electronic Science and Technology of China, Mianyang, China; 2 Department of Vascular Surgery, Mianyang Central Hospital, School of Medicine, University of Electronic Science and Technology of China, Mianyang, China; 3 Transfusion Department, Mianyang Central Hospital, School of Medicine, University of Electronic Science and Technology of China/NHC Key Laboratory of Nuclear Technology Medical Transformation (Mianyang Central Hospital), Mianyang, China

**Keywords:** autologous platelet-rich gel, venous malformation, refractory ulcer, growth factors, case report

## Abstract

Platelet-rich plasma (PRP) contains a high concentration of growth factors that promote angiogenesis, granulation-tissue formation and re-epithelialization, thereby significantly accelerating wound healing. Autologous platelet-rich gel (APG), a gelled form of PRP, provides a sustained release of these factors within a fibrin scaffold. We report a 25-year-old woman who developed a refractory plantar ulcer with tendon exposure after combined absolute-ethanol sclerotherapy and surgical excision of a congenital venous malformation of the left foot. Reflux of the sclerosant produced microcirculatory compromise, followed by full-thickness skin necrosis. Because the plantar skin is under high tension and has a relatively poor blood supply, conventional skin grafting was deemed unlikely to succeed. APG was therefore employed as a salvage treatment. After three applications, the ulcer area had decreased by 95%, and complete epithelialization was achieved within 1 month without scar contracture. Both functional and cosmetic outcomes were excellent. This case illustrates that APG promotes granulation and re-epithelialization of intractable ulcers following sclerotherapy for venous malformations, which reducing the need for grafting and representing a valuable adjunct for wounds with tendon exposure and compromised perfusion.

## Introduction

Peripheral venous malformations (PVMs) are congenital low-flow vascular anomalies characterized by dilated venous sinusoids and aberrant venous channels (Van Damme et al., 2020). Sclerotherapy with agents such as absolute ethanol is a first-line treatment, yet ulceration occurs in up to 18.7% of cases, with higher risks in plantar regions due to high skin tension and poor vascularity (Zhang et al., 2013). Such wounds often exhibit impaired microcirculation, recurrent infections, and growth factor deficiencies, leading to delayed healing. Traditional skin grafting has limited success here (Brem et al., 2007), necessitating advanced regenerative approaches.

Autologous platelet-rich plasma (PRP), concentrated via density gradient centrifugation, typically contains platelets at 3–7 times baseline levels (Hesseler and Shyam, 2019). Activated PRP releases growth factors, including platelet-derived growth factor (PDGF), transforming growth factor β (TGF-β), epidermal growth factor (EGF) and antimicrobial peptides, creating a sustained-release system that promotes tissue repair (Cecerska-Heryc et al., 2022). The addition of calcium chloride and thrombin yields platelet-rich gel (APG), which forms a fibrin scaffold that conforms to wound contours and facilitates prolonged factor release (Bakadia et al., 2023). APG also supports hemostasis, space occupancy, and cell adhesion, enhancing angiogenesis and collagen deposition (Gruber, 2000).

Substantial evidence supports the efficacy of PRP in chronic wounds, particularly in diabetic foot ulcers (DFUs). A meta-analysis of 12 randomized controlled trials (RCTs) showed PRP significantly shortened healing time and increased healing rates by 2.72-fold (Li et al., 2024). In DFUs, PRP achieved 66.7% complete healing at 6 months, reduced wound size from 2.17 cm to 0.4 cm, and accelerated healing by 3.21 weeks without increased infection or amputation risks (OuYang et al., 2023; Izzo et al., 2023). Furthermore, *Wang* et al. confirmed that PRP increased the proportion of completely healed wounds and shortened the time to complete healing. For venous ulcers, PRP improved the epithelialized area and the percentage of healed wound area. In vitiligo, PRP demonstrated superior outcomes compared to controls in terms of the degree of improvement and mean repigmentation (Wang et al., 2023). While growth factor gels such as Becaplermin are employed in DFUs treatment, their therapeutic action is restricted to the delivery of a single recombinant PDGF-BB to stimulate granulation tissue formation. In contrast, APG releases multiple growth factors (PDGF, TGF-β and EGF) simultaneously, thereby achieving multi-target, synergistic regulation of the healing process (Lacci and Dardik, 2010). In summary, PRP offers the combined benefits of accelerating healing, increasing healing rates, and conferring no additional risks across a variety of chronic refractory wounds. However, reports on APG in PVM-related plantar ulcers are lacking.

This case report highlights the success of APG in treating a plantar ulcer with tendon exposure post-PVM sclerotherapy, achieving 95% wound reduction and full epithelialization within 1 month. This approach overcomes anatomical constraints and avoids secondary surgery, offering new insights for microcirculation-impaired ulcers.

## Case description

**FIGURE 1:**
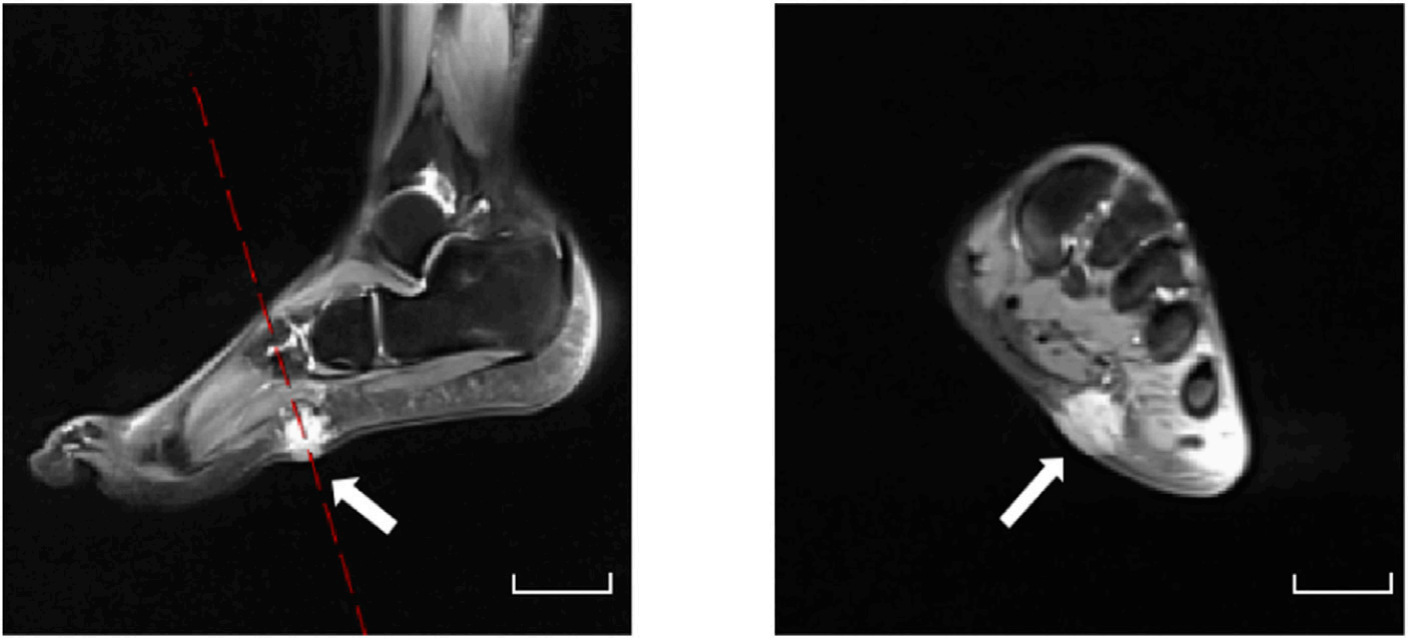
Plantar MRI. Pre-treatment MRI of the left plantar region showing a lobulated subcutaneous lesion (4 × 3 cm) along the lateral plantar aspect, isointense on T1-WI and hyperintense on fat-suppressed T2-WI. Scale bars, 5 cm.

A 25-year-old woman first presented a painful, progressively enlarging mass on the sole of her left foot 20 years ago, which was associated with overlying erythema. Non-contrast MRI revealed a 4 × 3 cm lobulated subcutaneous lesion along the lateral plantar aspect that was isointense on T1-weighted imaging (T1-WI) and hyperintense on fat-suppressed T2-WI (Figure 1). Based on the MRI findings, hemangioma and arteriovenous malformation were ruled out, and the final diagnosis was left lower-limb PVM.

**FIGURE 2:**
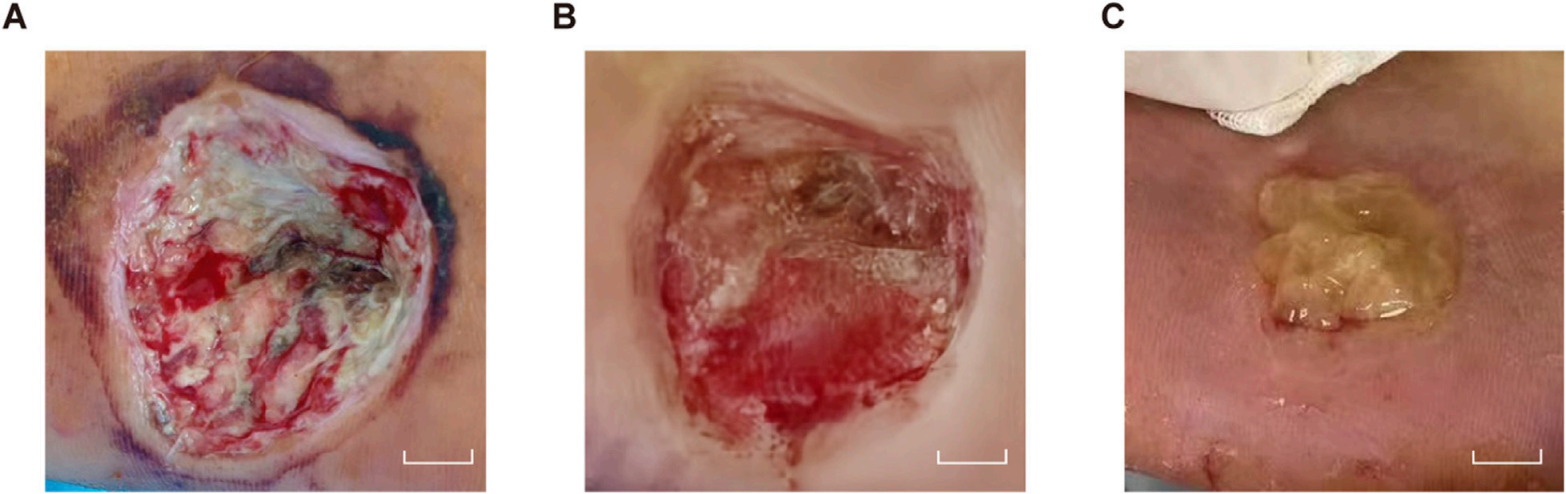
Pre-APG. Clinical photographs before APG application. **(A)** Full-thickness necrotic ulcer with tendon exposure. Scale bars, 1 cm. **(B)** Granulation after initial wound care. Scale bars, 1 cm. **(C)** Preparation of APG.

**FIGURE 3:**
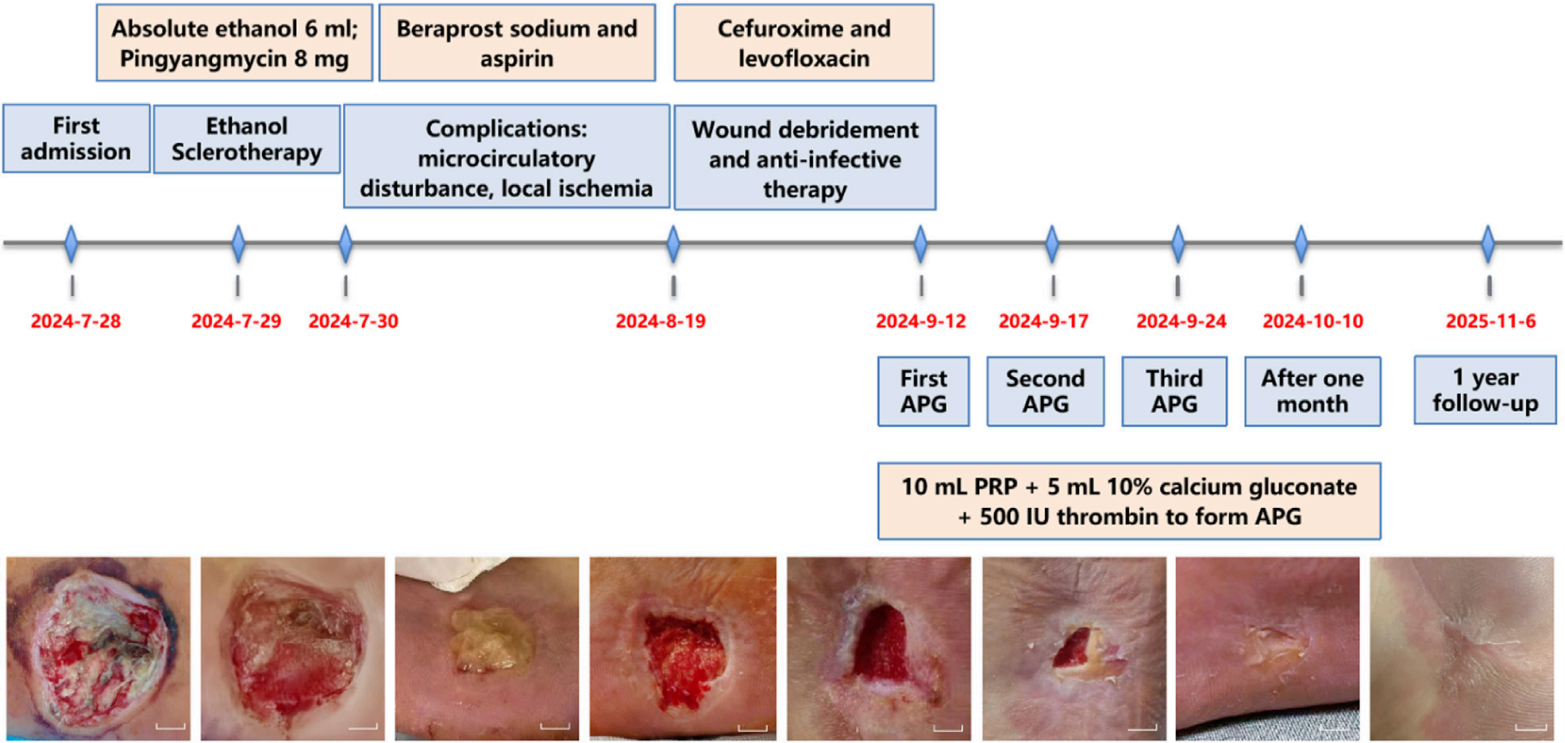
Clinical treatment timeline chart. This figure illustrates the treatment flowchart from admission through discharge and one-year follow-up, clearly demonstrating the changes in wound healing. Scale bars, 1 cm.

The patient underwent surgical excision combined with absolute ethanol sclerotherapy, during which 6 mL of absolute ethanol was administered. Postoperative care included oxygen therapy, continuous electrocardiographic monitoring. Pharmacological management with anti-inflammatory and analgesic agents, and papaverine administration to improve microcirculation. Unfortunately, reflux of the sclerosant caused peripheral arterial spasm and thrombosis, which resulted in local circulatory impairment. Postoperatively, a 4.5 cm × 3.5 cm × 1.5 cm full-thickness necrotic ulcer with exposed tendon developed (Figure 2A). At this point, the patient presented with pain and numbness in the left foot, accompanied by dusky red discoloration of the plantar skin and decreased skin temperature. The treatment regimen included anticoagulant therapy with beraprost sodium and aspirin, combined with hyperbaric oxygen therapy to improve tissue ischemia and hypoxia through vasodilation. The severe local infection was aggressively managed with a combination of cefuroxime and levofloxacin to combat the infection. Subsequent wound care included the application of silver-ion dressing for its antibacterial properties and alginate dressing to absorb purulent exudate, with daily dressing changes (Figure 3). Following this systematic therapy, the necrotic margins became clearly demarcated with granulation tissue formation, reducing the defect size to 2.5 cm × 2.0 cm × 1.0 cm (Figure 2B). Given the compromised vascular supply and elevated mechanical tension in the plantar region, skin grafting was deemed to have a low likehood of success. Consequently, APG therapy was initiated as an alternative reconstructive strategy.

**FIGURE 4:**
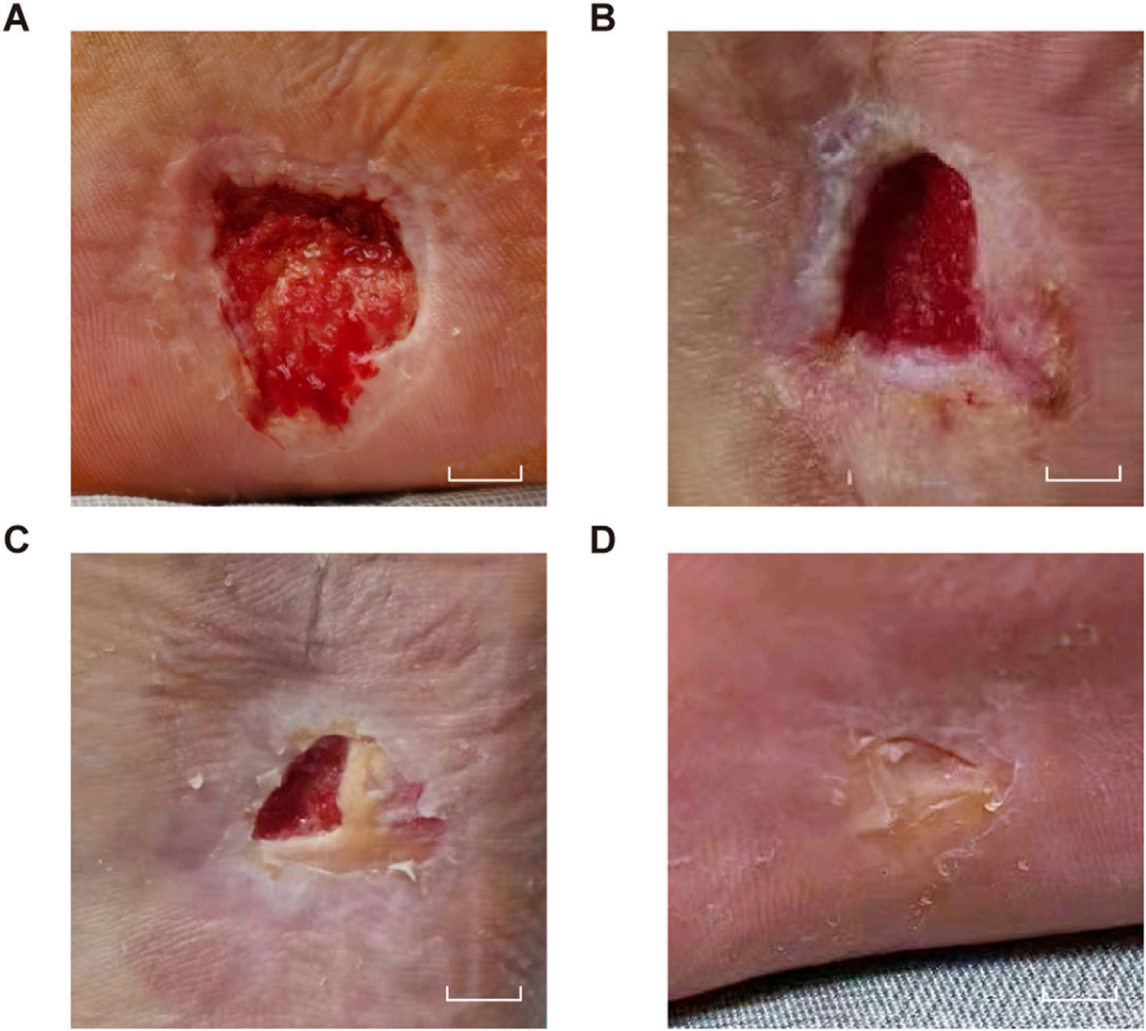
Post-APG. Sequential wound healing after APG. **(A)** Week 1. **(B)** Week 2. **(C)** Week 3. **(D)** Complete epithelialization at 1 month. Scale bars, 1 cm.

Laboratory tests revealed significantly abnormal postoperative coagulation function, with prolonged PT/APTT and decreased fibrinogen (Table 1). Additionally, testing performed 1 day prior to PRP collection showed Hb 118 g/L, RBCs 4.01 × 10^12^/L, and PLT 160 × 10^9^/L, with a plateletcrit of 0.21%, all meeting the criteria for PRP preparation. Using an MCS®+ED cell-separator (Haemonetics, United States), 500 mL of autologous venous blood was processed to yield 50 mL of concentrated PRP, which was then aseptically aliquoted into five individual pouches via a closed-system dock and stored at −80 °C. For each wound-care session, one pouch (10 mL) was mixed with 5 mL 10% calcium gluconate and 500 IU thrombin to form moldable APG (Figure 2C). Under strict asepsis, the APG was evenly applied to the ulcer base, covered with silver-impregnated antimicrobial dressing and petroleum gauze, then fixed with an elastic bandage under mild compression. All preparations strictly followed an identical standard operating procedure to ensure inter-batch consistency. Treatments were repeated every 7 days and three APG applications were completed in total. After the first week, the wound measured 1.3 × 1.0 × 0.5 cm (Figure 4A). In the second week, it measured 0.8 × 0.5 × 0.3 cm (Figure 4B). By the third week, it measured 0.7 × 0.5 × 0.1 cm (Figure 4C). One month after the final APG treatment, the wound was re-epithelialized and the plantar appearance and function were well restored (Figure 4D).

**TABLE 1:** Laboratory parameter changes.

Date/Parameter	7/28/2024	7/30/2024	8/19/2024	9/11/2024
PT (s)	12.2	13.3	12.0	12.4
APTT (s)	26.2	33.3	28.4	27.4
FIB (g/L)	2.08	1.95	2.77	2.00
LYM (×10^9^/L)	1.51	0.34	2.22	1.64
NEU (×10^9^/L)	1.35	8.00	4.63	2.53
PLT (×10^9^/L)	136	118	161	160

PT: Prothrombin Time; APTT: Activated Partial Thromboplastin Time; FIB: Fibrinogen; LYM: Lymphocyte; NEU: Neutrophil; PLT: Platelet.

**FIGURE 5:**
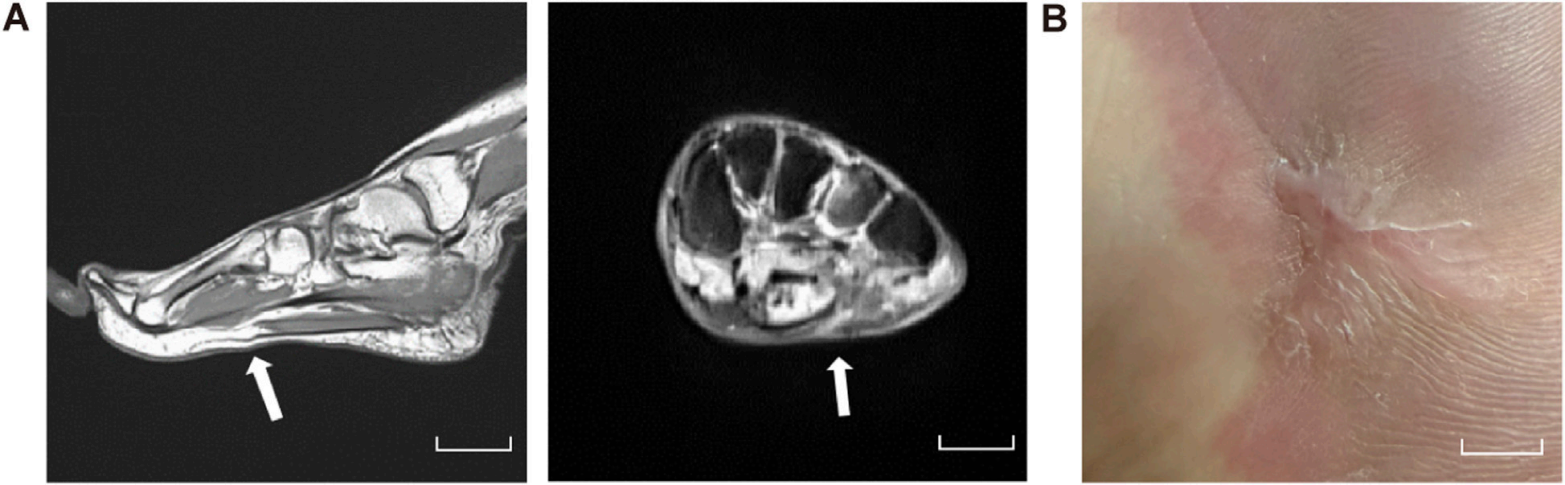
At the 1-year follow-up. **(A)** MRI of the left plantar foot. Scale bars, 5 cm. **(B)** Latest figure of wound healing. Scale bars, 1 cm.

Throughout the treatment period, no significant adverse reactions were observed. The patient’s pain and pruritus symptoms were significantly improved, with concomitant gradual recovery of sensory and motor functions in the left foot. Psychological assessment indicated a significant reduction in anxiety and depressive symptoms. At the 1-year follow-up, imaging demonstrated complete resolution of the plantar mass (Figure 5A) and full epithelialization of the wound (Figure 5B). Motor function had fully recovered, gait analysis revealed no abnormalities, and joint range of motion as well as muscle strength had returned to normal. The patient has since resumed occupational duties with no signs of recurrence, and reported a treatment satisfaction score of 10 out of 10 (Table 2).

**TABLE 2:** APG treatment and prognostic assessment scale.

APG Treatment Assessment Scale							
Week from APG	Date	VAS (rest 0–10)	VAS (weight-bearing 0–10)	Pain-free walking distance (m)	Wound volume (cm³)	HADS (0–21)	Satisfaction (0–10)
0	9/12/2024	4	5	0	2.5 ×2.0×1.0	13	4
1	9/17/2024	3	4	0	1.3×1.0×0.5	9	4
2	9/24/2024	1	2	≤50	0.8×0.5×0.3	8	5
3	10/10/2024	0	1	≤100	0	5	6
8	11/18/2024	0	0	≥500	0	3	8
52	11/6/2025	0	0	≥500	0	2	10

VAS: Visual Analogue Scale; HADS: Hospital Anxiety and Depression Scale.

## Discussion

Ulcers are caused by ischemia, pressure, or metabolic diseases, leading to impaired blood supply and disrupted repair processes. The core mechanism involves dysregulated healing, characterized by persistent inflammation, dysfunctional tissue-repairing cells, imbalanced extracellular matrix turnover, and inadequate angiogenesis (Jeffcoate et al., 2024). These factors, compounded by infection and a hypoxic microenvironment, prevent normal re-epithelialization and remodeling. A wound that shows no reduction after 4–6 weeks is clinically termed a “non-healing wound” (Zhao et al., 2016). This case demonstrates that APG supported granulation and epithelialization of therapy-refractory ulcers after PVM sclerotherapy, thereby reducing the need for grafting. It represents a particularly useful strategy for wounds with tendon exposure and compromised perfusion.

The ulcer in this case precipitated by retrograde leakage of absolute ethanol into the microcirculation, which caused arteriolar spasm and microthrombosis, thereby compromising regional perfusion. Beraprost sodium and aspirin were therefore administered to dilate microvessels, inhibit platelet aggregation, and restore distal blood flow. Critically, no exogenous granulation-promoting agents were employed during the treatment period. Thus, the observed neovascularization and accelerated wound healing are primarily attributed to the APG. However, given its single-case design, this study cannot exclude the possibility that the efficacy of APG was confounded by synergistic interventions such as debridement, silver ion dressings, and vasodilators. Controlled studies are needed to validate its independent therapeutic effect.

Compared to existing regenerative therapies, APG demonstrates distinct advantages. Unlike bioengineered skin substitutes, its autologous origin avoids immune rejection and offers greater integration potential in ischemic wounds. Compared to single-factor growth factor gels, APG provides a coordinated release of multiple growth factors, mimicking a more complete physiological healing process (Kawabata et al., 2023; Lana et al., 2023). Most importantly, the high platelet and fibrin content of APG ensures a sustained release of growth factors that enhance angiogenesis, collagen deposition and tissue regeneration (Wu et al., 2024). Concurrently, its fibrin component forms a three-dimensional scaffold that supports cell migration. In challenging ischemic tendon-exposed wounds, this scaffold additionally serves as a critical protective barrier against desiccation and infection (Ceccarelli and Putnam, 2014). Thus, APG transforms the single rapid-release profile of PRP into a sustained release mode that aligns with the physiological healing process. This characteristic enables prolonged regulation of repair phases such as inflammatory response, angiogenesis, and re-epithelialization (Song et al., 2023). By integrating the triple advantages of autologous safety, multi-factor synergy, and scaffold support, APG represents a compelling therapeutic strategy for complex wound repair.

Although PRP is well established in DFUs, mounting evidence supports its safety and efficacy in other ulcer types. Endoscopic PRP application for bleeding peptic ulcers significantly improves hemostasis and reduces re-bleeding rates (Xu et al., 2022). Similarly, intrauterine PRP infusion after hysteroscopic adhesiolysis has been shown to decrease adhesion recurrence and enhance endometrial thickness and menstrual recovery (Tang et al., 2023). PVM is characterized by dilated, thin-walled, valveless venous channels most commonly affecting the extremities and head/neck (Strauss et al., 2022). Ethanol sclerotherapy, the mainstay treatment, can cause extravasation or excessive micro-arteriolar occlusion, resulting in ischemic necrosis and ulceration, with ethanol reported to have the highest associated ulcer rate (Bouwman et al., 2020). Current management relies on conservative dressings or skin grafting.

Compared with traditional approaches, the sustained multi-factor release and fibrin scaffold provided by APG significantly accelerated granulation tissue proliferation and re-epithelialization, thereby achieving 95% wound closure and avoiding potential secondary trauma and functional impairment associated with skin grafting. The treatment successfully preserved the plantar weight-bearing structure and joint mobility, enabling the patient to restore normal gait and resume occupational activities. Furthermore, this approach shortened the overall treatment course, reduced the burden of frequent dressing changes and potential repeated surgeries, and demonstrated its potential as an efficient and cost-effective therapeutic option.

In conclusion, APG proved highly effective for a plantar ulcer with tendon exposure secondary to PVM sclerotherapy. By continuously releasing growth factors and improving local perfusion, APG promotes granulation and epithelialization, preserving plantar function. The procedure is straightforward to perform and safe, supporting its case for further validation and wider adoption in the management of similar complex wounds.

## Limitation

The single-case design limits the evidence level, and the definitive efficacy of APG for sclerotherapy-induced ulcers requires validation through large-scale cohort studies and randomized controlled trials. Furthermore, the lack of long-term follow-up data limits the systematic assessment of ulcer recurrence risk and long-term functional prognosis. Additionally, the absence of histopathological examination (e.g., HE staining and immunohistochemical analysis) precludes characterizing wound microenvironment features and elucidation of APG’s cellular-molecular mechanisms. Future research should conduct multicenter prospective clinical trials that integrating histomorphologic and molecular marker analyses. This approach will elevate the quality of evidence, reveal underlying mechanistic pathways, and ultimately providing high-level evidence for optimize treatment paradigms for microcirculation-impaired wounds.

## Conclusion

This report documents the first successful use of APG to close a therapy-refractory plantar ulcer with exposed tendon following sclerotherapy. APG achiveved a 95% reduction in wound area with complete epithelialization within 4 weeks, obviating the need for skin grafting in a region of poor vascularity and high tension. The outcome confirms that APG can safely accelerate granulation and re-epithelialization in microcirculation-compromised, wounds with tendon exposing. Despite the limitations inherent in a single-case report, APG constitutes a promising, low-morbidity adjunct for similar complex wounds and merits validation in larger controlled trials.

## Data Availability

The original contributions presented in the study are included in the article/supplementary material, further inquiries can be directed to the corresponding author.
